# Multicentered hydrogen bonding in 1-[(1-de­oxy-β-d-fructo­pyranos-1-yl)aza­nium­yl]cyclo­pentane­carboxyl­ate (‘d-fructose-cyclo­leucine’)

**DOI:** 10.1107/S2056989019009253

**Published:** 2019-07-02

**Authors:** Valeri V. Mossine, Charles L. Barnes, Thomas P. Mawhinney

**Affiliations:** a Department of Biochemistry, University of Missouri, Columbia, MO 65211, USA; b Department of Chemistry, University of Missouri, Columbia, MO 65211, USA; c Department of Biochemistry, University of Missouri, Columbia, MO 65211, U.S.A.

**Keywords:** crystal structure, fructosamine, Amadori rearrangement, cyclo­leucine, hydrogen bonding, Hirshfeld surface analysis

## Abstract

The title compound, C_12_H_21_NO_7_, (**I**), is conformationally unstable; the predominant form present in its solution is the β-pyran­ose form (74.3%), followed by the β- and α-furan­oses (12.1 and 10.2%, respectively), α-pyran­ose (3.4%), and traces of the acyclic carbohydrate tautomer. In the crystalline state, the carbohydrate part of (**I**) adopts the ^2^ *C*_5_ β-pyran­ose conformation, and the amino acid portion exists as a zwitterion, with the side chain cyclo­pentane ring assuming the *E*_9_ envelope conformation. All heteroatoms are involved in hydrogen bonding that forms a system of anti­parallel infinite chains of fused *R*_3_^3^(6) and *R*_3_^3^(8) rings. The mol­ecule features extensive intra­molecular hydrogen bonding, which is uniquely multicentered and involves the carboxyl­ate, ammonium and carbohydrate hy­droxy groups. In contrast, the contribution of inter­molecular O⋯H/H⋯O contacts to the Hirshfeld surface is relatively low (38.4%), as compared to structures of other d-fructose-amino acids. The ^1^H NMR data suggest a slow rotation around the C1—C2 bond in (**I**), indicating that the intra­molecular heteroatom contacts survive in aqueous solution of the mol­ecule as well.

## Abstract 2

The mol­ecule is a zwitterion and features a strong multicentered intra­molecular hydrogen bonding involving the carboxyl, amino, and anomeric hydroxyl groups. It adopts the ^2^ *C*_5_ β-pyran­ose conformation, which also the dominant form present in its solution.

## Chemical context   

d-Fructosamine derivatives are products of non-enzymatic condensation reactions between d-glucose and biomolecules containing free aliphatic amino groups, such as amino acids, proteins, amino­phospho­lipids, or biogenic amines (Mossine & Mawhinney, 2010 ▸). d-Fructosamines are thus present in all living systems and in foods. For instance, in healthy humans, about 5% of plasma proteins are decorated with fructosamine residues, while dietary intake of d-fructosamines, primarily in the form of *N*_∊_-(1-de­oxy-d-fructos-1-yl)-l-lysine, has been estimated at 1 g per day. Although the normal physiological functions of d-fructosamines are not understood, a number of bacterial, fungal, and mammalian carbohydrate-processing enzymes (Wu & Monnier, 2003 ▸; Van Schaftingen *et al.*, 2012 ▸), transporters (Marty *et al.*, 2016 ▸), and lectins (Mossine *et al.*, 2008 ▸) can recognize d-fructosamine, thus implying the participation of this structure in metabolic and signaling processes. Biomedical research has suggested the involvement of d-fructosamines in the development of diabetic complications (Wu & Monnier, 2003 ▸), bacterial infections (Ali *et al.*, 2014 ▸), and cancer (Malmström *et al.*, 2016 ▸). We and others (Mossine *et al.*, 2010 ▸; Rabinovich *et al.*, 2006 ▸) have demonstrated the efficacy of synthetic d-fructosamine derivatives as blockers of galectins, a family of tumor-associated lectins. In this context, several structure determinations of biologically active fructosamines have previously been undertaken (Mossine *et al.*, 2007*a* ▸,*b* ▸, 2009 ▸, 2018 ▸).
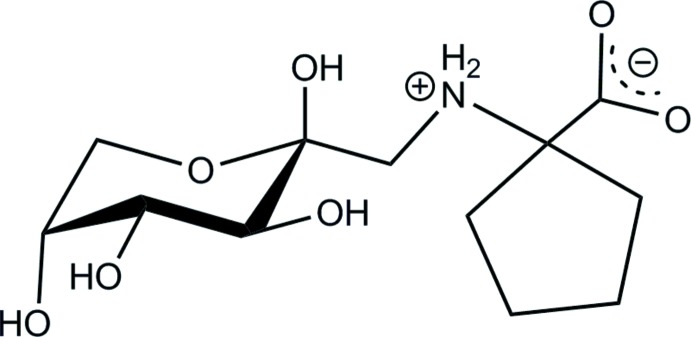


As a part of our search for efficient blockers of galectins-1, −3 and −4, we have prepared d-fructose-cyclo­leucine (**I**), a structural analog of the galectin inhibitor d-fructose-l-leucine (Mossine *et al.*, 2008 ▸). Here we report on the mol­ecular and crystal structures of (**I**), with an emphasis on the hydrogen-bonding patterns in the structure.

## Structural commentary   

**Figure 1:**
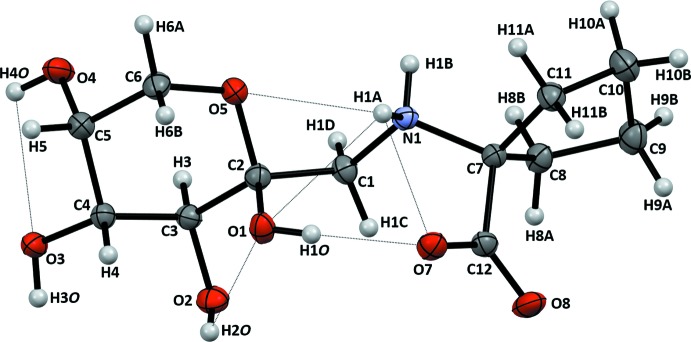
The title compound (**I**) with the atomic numbering and displacement ellipsoids drawn at the 50% probability level. Intra­molecular N—H⋯O and O—H⋯O inter­actions are shown as dotted lines.

**Figure 2:**
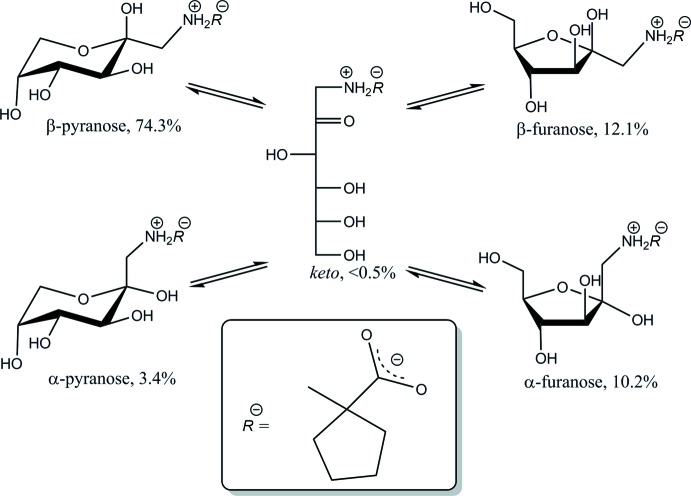
Tautomeric equilibrium in aqueous solution of d-fructose-cyclo­leucine at 293 K and pH 6, as determined by ^13^C NMR.

The mol­ecular structure and atomic numbering are shown in Fig. 1 ▸. The title compound, (**I**), crystallizes in the monoclinic space group *P*2_1_, with two equivalent mol­ecules per unit cell. The mol­ecule may be considered as a conjugate of a carbohydrate, 1-amino-1-de­oxy-d-fructose, and an amino acid, 1-amino­cyclo­pentane-1-carb­oxy­lic acid, which are joined through the common amino group. The β-d-pyran­ose ring of the carbohydrate portion exists in the ^2^ *C*_5_ or 1C(D) chair conformation, with puckering parameters *Q* = 0.5763 Å, *θ* = 172.71°, and *φ* = 248.80°. These parameters correspond to a conformation with the lowest energy possible for fructose (French *et al.*, 1997 ▸). The bond distances and the valence angles are close to the average values for a number of crystalline pyran­ose structures (Jeffrey & Taylor, 1980 ▸). In an aqueous solution of (**I**), the β-d-pyran­ose anomer dominates the tautomeric equilibrium (Fig. 2 ▸), at 74.3%, as follows from its ^13^C NMR spectrum (Table 1 ▸). The acyclic forms are not readily detectable because of their low populations; their presence is suggested based on literature evidence available for other fructosamine derivatives (Table 1 ▸). In the ^1^H NMR spectrum of the major anomer, the vicinal proton–proton coupling constants *J*_3,4_ = 9.8 Hz and *J*_4,5_ = 3.4 Hz indicate that atom H4 is in a *trans* disposition to H3 and in a *gauche* disposition to H5. Hence, the predominant conformation of d-fructose-cyclo­leucine in solution is also ^2^ *C*_5_ β-d-fructo­pyran­ose.

The amino acid portion of the mol­ecule is in the zwitterionic form, with a positively charged tetra­hedral secondary ammonium nitro­gen atom and a negatively charged deprotonated carboxyl group. The side-chain cyclo­pentane ring is, by the atom numbering in Fig. 1 ▸, in the *E*_9_ (envelope on C9) or C_s_—C^γ^-*exo* conformation, with puckering parameters *Q* = 0.4220 Å, *φ* = 254.94°, and pseudorotational parameters (Rao *et al.*, 1981 ▸) *P* = 56.9° and *τ* = 43.7° for the C7—C8 bond.

**Figure 3:**
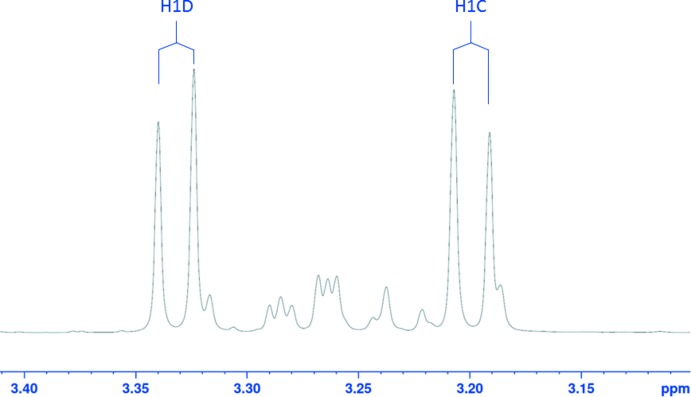
A portion of the ^1^H NMR spectrum of d-fructose-cyclo­leucine in D_2_O at 293 K containing four sets of signals for methyl­ene protons H1*C* and H1*D*. The labeled set of two doublets belongs to the dominant β-d-fructo­pyran­ose anomer of (**I**). The smaller, unlabeled peaks are unresolved signals of H1*C* and H1*D* belonging to the α-pyran­ose, α- and β-furan­ose conformations of (**I**).

The ammonium group and all but one (O8) oxygen atoms are involved in intra­molecular hydrogen bonding (Table 2 ▸). At the centre of this system are heteroatom contacts between the conjugated carbohydrate and the amino acid portions of the mol­ecule, which involve the carboxyl­ate atom O7, the ammonium atom H1*A*, the pyran­ose ring atom O5, and the anomeric hydroxyl group O1—H1*O* (Fig. 1 ▸). Although the value of N1—H1*A*⋯O7 angle is 99.4°, the distance N1⋯O7 is 2.702 (2) Å, short enough for this heteroatom contact to qualify as a strong hydrogen bond. Then the central motif of the intra­molecular hydrogen-bonded structure can be described in terms of a compact ring ***S*** **_2_^2^(4)** pattern represented by the four-atom O7⋯H1*A*⋯O1—H1*O*⋯O7 cycle. In the ^1^H NMR spectrum of (**I**), two protons, H1*C* and H1*D*, which are attached to C1, produce two distinct signals at 3.332 and 3.199 ppm, with *J*_1C,1D_ = −12.8 Hz (Fig. 3 ▸). The non-equivalence of these protons indicates restricted rotation around the C1—C2 and C1—N1 bonds, thus suggesting the intra­molecular hydrogen bonding retains this structure in solution.

## Supra­molecular features   

**Figure 4:**
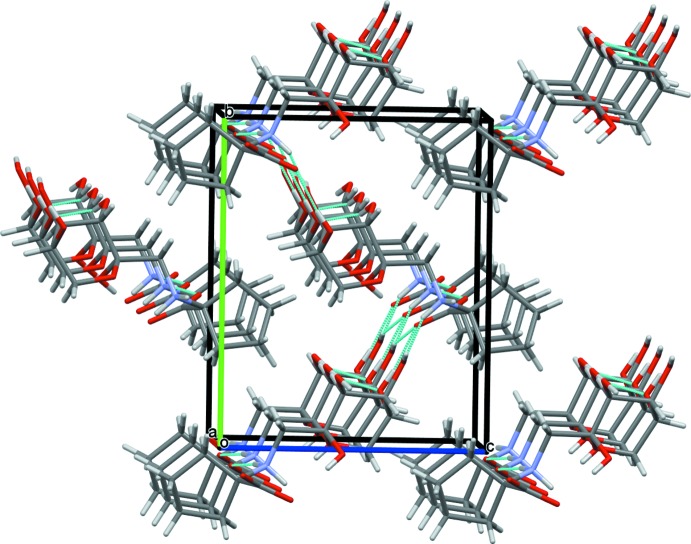
The mol­ecular packing in (**I**). A view of the unit-cell contents shown in projection down the *a* axis. Color code for crystallographic axes: red − *a*, green − *b*, blue − *c*. Hydrogen bonds are shown as cyan dotted lines.

**Figure 5:**
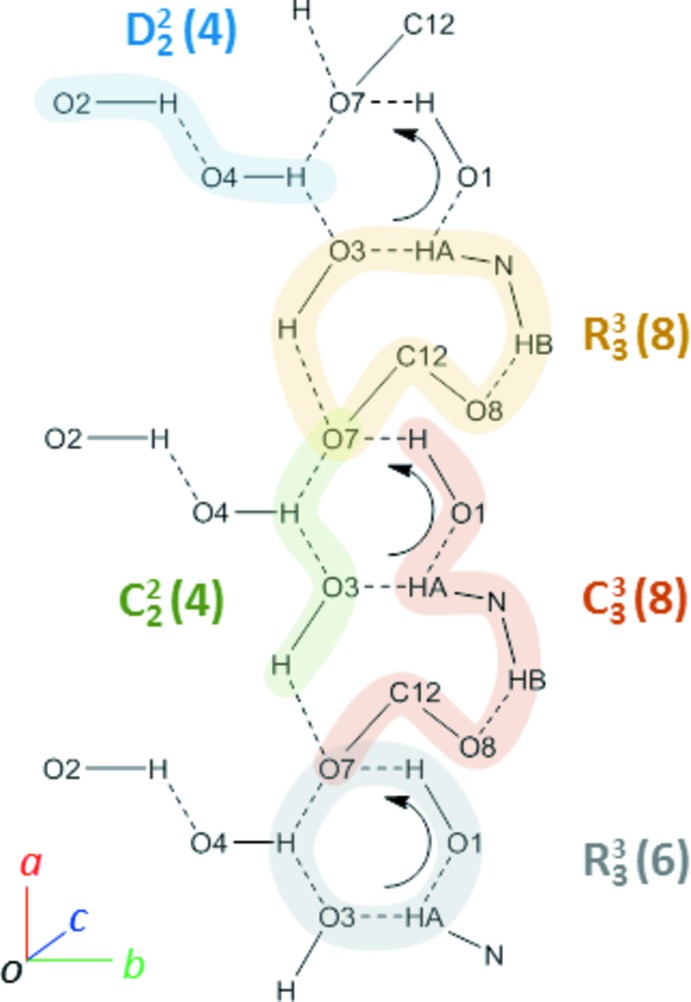
Hydrogen-bonding patterns in the crystal structure of (**I**), as viewed down the *c* axis. Weakly directional intra­molecular hydrogen bonds are excluded from the figure.

**Figure 6:**
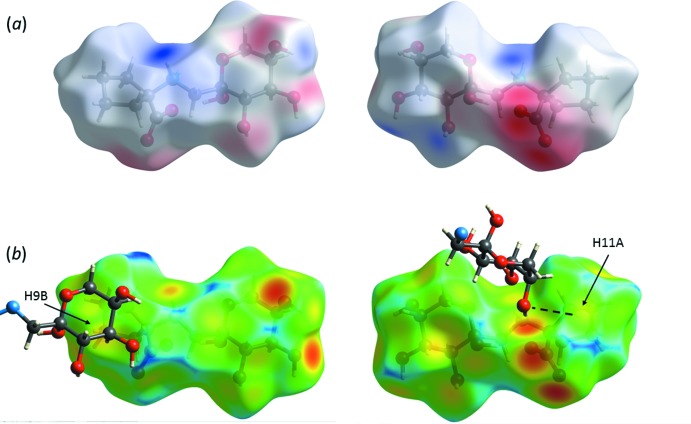
Views of the Hirshfeld surface for (**I**) mapped over: (*a*) the electrostatic potential in the range −0.156 to +0.261 a.u. with the red and blue colors representing the distribution of the negative and positive electrostatic potential, respectively; (*b*) the *d*_e_ function, in the range 0.674 to 2.424 Å, calculated for the external contact atoms in the crystal. The mol­ecular fragments involved in short C—H⋯O inter­actions are shown; these allegedly stabilize the cyclo­pentane ring conformation in crystalline (**I**).

The crystal packing of (**I**) features infinite chains of anti­parallel hydrogen bonds running along the *a*-axis direction (Fig. 4 ▸). The basic hydrogen-bonding patterns are depicted in Fig. 5 ▸ and include two rings, ***R*** **_3_^3^(6)** and ***R*** **_3_^3^(8)**, and a small finite chain ***D*** **_2_^2^(4)**. Alternatively, the fused rings pattern can be described in terms of two chains, ***C*** **_2_^2^(4)** and ***C*** **_3_^3^(8)**. The ammonium proton H1*A* is involved in a rare five-centered hydrogen bond, involving three weakly directional intra­molecular contacts with O1, O5, and O7 (at distances of 2.59, 2.43, and 2.40 Å, respectively) and one inter­molecular, shorter (2.00 Å distance) bond with O3. The carboxyl atom O7 is also involved in an unusual multicenter hydrogen bond, by coord­inating four surrounding protons at reasonably short distances, 1.97–2.40 Å (Tables 2 ▸ and 3 ▸). This multicentered character of the short heteroatom contacts implies a significant contribution of the electrostatic component (Tao *et al.*, 2017 ▸) to the inter­action, apparently between the positively charged ammonium group and the negatively charged carboxyl atom O7 (Fig. 6 ▸). Indeed, the C12—O7 bond [1.269 (3) Å] is significantly longer than the C12—O8 distance [1.243 (3) Å], suggesting a more polarized character of the former. This may be a consequence of highly differing heteroatom arrangements around the two carboxyl­ate oxygen atoms in the crystal. One, O7, is surrounded by four heteroatoms (O1, O3, O4, N1) at distances qualifying for hydrogen bonds, while O8 has only one heteroatom, N1, located at a short distance.

**Figure 7:**
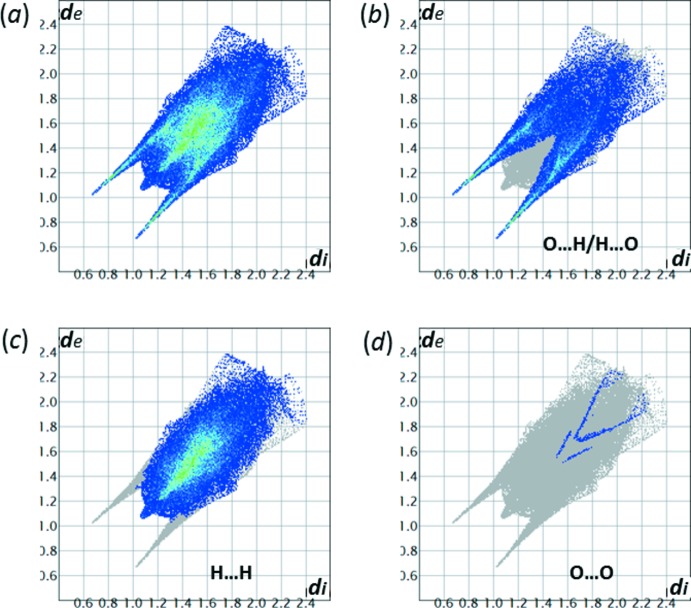
(*a*) The full two-dimensional fingerprint plot for (**I**) and those delineated into specific contacts: (*b*) O⋯H/H⋯O (38.5% contribution to the Hirshfeld surface); (*c*) H⋯H (60.9%); (*d*) O⋯O (0.6%).

The Hirshfeld surface analysis (Spackman & Jayatilaka, 2009 ▸) revealed that a major proportion of the inter­molecular contacts in crystal structure of (**I**) is provided by non- or low-polar H⋯H inter­actions (Fig. 7 ▸). Of note, there are three short inter­atomic contacts of the C—H⋯O type (Table 3 ▸, Fig. 6 ▸) that involve the cyclo­pentane ring and which may be responsible for conformational stabilization of the ring. In contrast, a number of published cyclo­leucine structures feature disordered conformations as a result of the ring pucker pseudorotation (Mallikarjunan *et al.*, 1972 ▸; Varughese & Chacko, 1978 ▸; Santini *et al.* 1988 ▸).

## Database survey   

Searches of SciFinder (2018 ▸) and the Cambridge Structural Database (2019 CSD release; Groom *et al.*, 2016 ▸) by both structure and chemical names returned no previous structural description of *N*-(1-de­oxy-β-d-fructo­pyranos-1-yl)-1′-amino­cyclo­pentane-1′-carb­oxy­lic acid or d-fructose-cyclo­leucine; thus the compound appears to be novel. Since the conformational instability of the d-fructosamine moiety determines the chemical reactivities and biological activities of d-fructosamine derivatives (Mossine & Mawhinney, 2010 ▸), we compared the structure of (**I**) with solved structures of other d-fructose-amino acids. The most closely related structures are d-fructose-2-amino­isobutyric acid (CCDC 1583254; Mossine *et al.*, 2018 ▸), d-fructose-glycine (CCDC 1307697; Mossine, Glinsky *et al.*, 1995 ▸), d-fructose-l-proline [CCDC 628806 and 628807 (Tarnawski *et al.*, 2007 ▸), 631528 (Mossine *et al.*, 2007*a* ▸)], and d-fructose-l-histidine (CCDC 622419; Mossine *et al.*, 2007*b* ▸). Although some fructosamine derivatives can crystallize as the β-furan­ose, *spiro*-bicyclic hemiketal, or acyclic *keto* tautomers (Mossine, Barnes *et al.*, 1995 ▸, 2009 ▸), all of the above-listed d-fructose-amino acids adopt the ^2^ *C*_5_ β-pyran­ose conformation and exist as zwitterions, with the intra­molecular hydrogen-bonding central pattern localized around the ammonium group and involving the carboxyl­ate and one hydroxyl group donated by the carbohydrate moiety. This hydrogen-bonded conjugation between the amino acid zwitterion bridge and the β-pyran­ose provides for conformational stability around the C1—C2 bond in solutions of d-fructose-amino acids. The staggered *gauche–trans* conformation of the N1—C1—C2—O5 torsion, such as in (**I**), has also been observed in CCDC 1583254 (mol­ecule *A*; Mossine *et al.*, 2018 ▸), CCDC 631528 (Mossine *et al.*, 2007*a* ▸), and CCDC 622419 (Mossine *et al.*, 2007*b* ▸), while the *trans–gauche* conformation was observed in four other structures of d-fructose-amino acids (Table 4 ▸). However, none of these structures, except (**I**), features the cyclic motif of intra­molecular multicentered hydrogen bonding (Fig. 1 ▸), which is supported by a unique direct inter­action between the carbohydrate anomeric hydroxyl donor, O1—H1*O*, and the carboxyl­ate acceptor, O7. In total, there are six intra­molecular short heteroatom contacts in the structure of (**I**), more than in any other d-fructose-amino acid structure known to date. Such effect of the ‘inter­nalization’ of hydrogen bonding in (**I**) is also revealed in a comparative analysis of the fingerprint plots (Fig. 7 ▸) that are based on the calculations of Hirshfeld surfaces (Spackman & Jayatilaka, 2009 ▸) and delineated into the O⋯H/H⋯O inter­molecular contacts in the crystal structure of (**I**). The relative abundance of these contacts in structures of d-fructose-amino acids decreases with an increase in the number of intra­molecular hydrogen bonds; this trend is clearly revealed by the data presented in Table 4 ▸. The significant difference between the carboxyl­ate C—O lengths of 0.026 Å in (**I**) is comparable to the respective bond-length differences noted in other fructose-amino acid structures, including CCDC 1583254 (0.022 Å in mol­ecule *B*; Mossine *et al.*, 2018 ▸) and CCDC 622419 (0.021 Å; Mossine *et al.*, 2007*b* ▸). In the latter two structures, the carboxyl­ate oxygen atoms are involved in close heteroatom contacts unequally, although not to the extent observed in (**I**).

## Synthesis and crystallization   

Cyclo­leucine (2.6 g, 0.02 mol), d-glucose (9 g, 0.05 mol), and sodium acetate (0.82 g, 0.01 mol) were dissolved in 100 mL of a methanol/glycerol (3:1) mixture and refluxed for 3 h. The reaction progress was monitored by TLC on silica. The reaction mixture was diluted with 900 mL of water and passed through a column charged with 80 mL of Amberlite IRN-77 (H^+^-form). The target compound was then eluted with 0.2 *M* pyridine, and fractions containing pure (**I**) were pooled and evaporated. The residue was redissolved in 100 mL of water, decolorized with 0.5 g of charcoal and evaporated to a syrup. The latter was dissolved in 30 mL of ethanol and made nearly cloudy with the dropwise addition of acetone. Crystallization occurred within a week at room temperature. Yield 3.4 g (58%, based on the starting cyclo­leucine).

Major β-pyran­ose tautomer peaks (ppm) in ^13^C NMR spectrum in D_2_O: 179.82 (C12); 98.34 (C2); 76.30 (C7); 72.33 (C4); 72.20 (C3); 71.80 (C5); 66.66 (C6); 52.89 (C1); 37.44, 37.40 (C8, C11); 28.00, 27.98 (C9, C10). See Table 1 ▸ for minor peaks assignments in the spectrum. Major signals (ppm) and resolved coupling constants (Hz) in the ^1^H NMR spectrum: 4.035 (*dd*, H6*B*); 4.017 (*m*, H5); 3.896 (*dd*, H4); 3.774 (*d*, H3); 3.771 (*dd*, H6*A*); 3.332 (*d*, H1*D*); 3.199 (*d*, H1*C*); 2.220 (*m*, 2H11); 1.955 (*m*, 2H8); 1.83 (*m*, 2H9 + 2H10); *J*_1C,1D_ = −12.8; *J*_3,4_ = 9.8; *J*_4,5_ = 3.4; *J*_5,6A_ = 1.3; *J*_6A,6B_ = −12.9.

## Refinement details   

Crystal data, data collection and structure refinement details are summarized in Table 5 ▸. Hydroxyl H atoms were located in difference-Fourier maps and were allowed to refine freely. Other H atoms were placed at calculated positions and treated as riding, with N—H = 0.91 Å, C—H = 0.99 Å (methyl­ene) or 1.00 Å (methine) and with *U*_iso_(H) = 1.2*U*_eq_ (methine or methyl­ene). As a result of the unrealistic value obtained for the Flack absolute structure parameter [−0.4 (4) for 1097 quotients; Parsons *et al.*, 2013 ▸], the absolute configuration of the pyran­ose ring system (2*R*,3*S*,4*R*,5*R*) was assigned on the basis of the known configuration for the starting compound d-glucose (McNaught, 1996 ▸).

## Supplementary Material

Crystal structure: contains datablock(s) I. DOI: 10.1107/S2056989019009253/eb2020sup1.cif

Structure factors: contains datablock(s) I. DOI: 10.1107/S2056989019009253/eb2020Isup2.hkl

CCDC reference: 1583255

Additional supporting information:  crystallographic information; 3D view; checkCIF report

## Figures and Tables

**Table 1:** Chemical shifts (p.p.m.) in the ^13^C NMR spectrum of (I) and the anomeric distribution of D-fructose-cyclo­leucine and structurally related mol­ecules in D_2_O at 293 K

Carbon	α-pyran­ose	β-pyran­ose	α-furan­ose	β-furan­ose	acyclic
C1	52.61	52.89	51.12	52.51	
C2	99.11	98.34	104.69	101.84	
C3	73.12	72.20	85.18	80.58	
C4	74.75	72.33	78.71	77.12	
C5	68.73	71.80	85.32	83.73	
C6	65.78	66.66	63.63	64.76	
Cα	75.99	76.30	76.14	76.24	
					
% for (**I**)	*3.4*	*74.3*	*10.2*	*12.1*	*< 0.5*
% for fructose^*a*^	*2.1*	*68.6*	*5.7*	*23.0*	*0.5*
% for FruLeu^*b*^	*4*	*72*	*12*	*12*	*< 1*
% for FruAib^*c*^	*3.0*	*75.6*	*10.1*	*10.4*	*< 0.7*
% for FruPro^*a*^	*4.2*	*64.8*	*12.9*	*16.9*	*1.2*

Notes: (*a*) Kaufmann *et al.* (2016 ▸); (*b*) Glinsky *et al.* (1996 ▸); (*c*) Mossine *et al.* (2018 ▸).

**Table 2:** Hydrogen-bond geometry (Å, °)

*D*—H⋯*A*	*D*—H	H⋯*A*	*D*⋯*A*	*D*—H⋯*A*
N1—H1*B*⋯O8^i^	0.91	1.80	2.704 (2)	174
N1—H1*A*⋯O3^ii^	0.91	2.00	2.784 (2)	143
N1—H1*A*⋯O1	0.91	2.59	2.980 (2)	107
O4—H4*O*⋯O3	0.85 (3)	2.41 (3)	2.746 (2)	105 (2)
O4—H4*O*⋯O7^iii^	0.85 (3)	2.09 (3)	2.845 (2)	149 (3)
O1—H1*O*⋯O7	0.80 (4)	1.97 (4)	2.769 (2)	178 (3)
O3—H3*O*⋯O7^iv^	0.84 (4)	1.99 (4)	2.796 (2)	162 (3)
O2—H2*O*⋯O4^v^	0.81 (3)	2.00 (3)	2.765 (2)	159 (3)

Symmetry codes: (i) 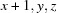; (ii) 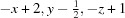; (iii) 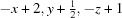; (iv) 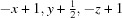; (v) 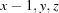.

**Table 3:** Suspected hydrogen bonds and short C—H⋯*A* contacts (Å, °)

*D*—H⋯*A*	*D*—H	H⋯*A*	*D*⋯*A*	*D*—H⋯*A*	Symmetry code
N1—H1*A*⋯O7	0.91	2.40	2.702 (2)	99	
N1—H1*A*⋯O5	0.91	2.43	2.698 (2)	97	
O2—H2*O*⋯O1	0.81 (3)	2.60 (4)	2.845 (2)	99	
C8—H8*A*⋯O8	0.99	2.47	2.810 (3)	100	
C9—H9*B*⋯O1	0.99	2.66	3.618 (3)	162	*x*, *y*, *z* − 1
C11—H11*A*⋯O3	0.99	2.65	3.416 (3)	134	−*x* + 2, *y* − 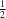 , −*z* + 1
C4—H4⋯O4	1.00	2.69	3.364 (3)	124	*x* − 1, *y*, *z*
C6—H6*A*⋯O1	0.99	2.67	3.430 (3)	134	*x* + 1, *y*, *z*

**Table 4:** Conformation, intra­molecular hydrogen bonding around the amino group, and contributions of the inter­molecular O⋯H/H⋯O contacts to the Hirshfeld surfaces in *N*-(β-D-fructo­pyranos-1-yl)-amino acids Hydrogen-bond selection criteria: *D*⋯*A* < 3.0 Å; H⋯*D* < 2.7 Å; *D*—H⋯*A* > 95°.

Structure	N—C1—C2—O5 torsion (°), conformation	Intra­molecular hydrogen bonds around the amino group	No. of intra/intermol­ecular hydrogen bonds	O⋯H/H⋯O contacts on Hirshfeld surface (%)
Fru-cycloLeu, (**I**)	+53.3 *gt*	N1—H1*A*⋯O1 (106°); N1—H1*A*⋯O5 (97°); N1—H1*A*⋯O7 (99°); O1—H1*O*⋯O7 (178°)	6/5	38.5
FruGly^*a*^	+165.5 *tg*	N1—H1*A*⋯O2 (140°); N1—H1*A*⋯O7 (104°)	2/6	51.6
FruAib (mol­ecule *A*)^*b*^	+64.7 *gt*	N1—H1*B*⋯O5 (110°); N1—H1*B*⋯O7 (107°)	3/5	44.0
FruAib (mol­ecule *B*)^*b*^	+176.8 *tg*	N1—H1*A*⋯O2 (145°); N1—H1*A*⋯O7 (100°)	3/5	45.9
FruPro·H_2_O^*d*^	+75.8 *gt*	N1—H1⋯O1 (109°); N1—H1⋯O7 (125°)	3/6	49.2
FruPro·2H_2_O^*d*^	+176.8 *tg*	N1—H1⋯O2 (140°); N1—H1⋯O7 (113°)	3/6	49.3
FruPro·MeOH^*d*^	+174.4 *tg*	N1—H1⋯O2 (139°); N1—H1⋯O7 (114°)	4/5	40.2
FruHis·H_2_O^*e*^	+60.7 *gt*	N1—H1*B*⋯O5 (100°); N1—H1*B*⋯O7 (102°); N1—H1*A*⋯O1 (108°)	5/7	41.2

Notes: (*a*) Mossine, Glinsky *et al.* (1995 ▸); (*b*) Mossine *et al.* (2018 ▸); (*c*) Mossine *et al.* (2007*a* ▸); (*d*) Tarnawski *et al.* (2007 ▸); (*e*) Mossine *et al.* (2007*b* ▸).

**Table 5:** Experimental details

Crystal data	
Chemical formula	C_12_H_21_NO_7_
*M*_r_	291.30
Crystal system, space group	Monoclinic, *P*2_1_
Temperature (K)	100
*a*, *b*, *c* (Å)	5.8052 (3), 11.9540 (6), 9.6135 (5)
β (°)	95.506 (1)
*V* (Å^3^)	664.05 (6)
*Z*	2
Radiation type	Mo *K*α
μ (mm^−1^)	0.12
Crystal size (mm)	0.55 × 0.25 × 0.10

Data collection	
Diffractometer	Bruker APEXII CCD area detector
Absorption correction	Multi-scan (*SADABS*; Krause *et al.*, 2015 ▸)
*T*_min_, *T*_max_	0.72, 0.99
No. of measured, independent and observed [*I* > 2σ(*I*)] reflections	4797, 2761, 2619
*R*_int_	0.017
(sin θ/λ)_max_ (Å^−1^)	0.641

Refinement	
*R*[*F* ^2^ > 2σ(*F* ^2^)], *wR*(*F* ^2^), *S*	0.029, 0.073, 1.03
No. of reflections	2761
No. of parameters	197
No. of restraints	1
H-atom treatment	H atoms treated by a mixture of independent and constrained refinement
Δρ_max_, Δρ_min_ (e Å^−3^)	0.23, −0.20
Absolute structure parameter	−0.4 (4)

Computer programs: *SMART* and *SAINT-Plus* (Bruker, 1998 ▸), *SHELXS97* (Sheldrick, 2008 ▸), *SHELXL2017/1* (Sheldrick, 2015 ▸), *Mercury* (Macrae *et al.*, 2008 ▸), *CIFTAB* (Sheldrick, 2008 ▸) and *publCIF* (Westrip, 2010 ▸).

## supplementary crystallographic information

### Crystal data

**Table:**

C_12_H_21_NO_7_	*F*(000) = 312
*M**_r_* = 291.30	*D*_x_ = 1.457 Mg m^−^^3^
Monoclinic, *P*2_1_	Mo *K*α radiation, λ = 0.71073 Å
*a* = 5.8052 (3) Å	Cell parameters from 3178 reflections
*b* = 11.9540 (6) Å	θ = 2.7–27.1°
*c* = 9.6135 (5) Å	µ = 0.12 mm^−^^1^
β = 95.506 (1)°	*T* = 100 K
*V* = 664.05 (6) Å^3^	Plate, colourless
*Z* = 2	0.55 × 0.25 × 0.10 mm

### Data collection

**Table:**

Bruker APEXII CCD area detector diffractometer	2619 reflections with *I* > 2σ(*I*)
ω scans	*R*_int_ = 0.017
Absorption correction: multi-scan (SADABS; Krause *et al.*, 2015)	θ_max_ = 27.1°, θ_min_ = 2.1°
*T*_min_ = 0.72, *T*_max_ = 0.99	*h* = −7→7
4797 measured reflections	*k* = −15→15
2761 independent reflections	*l* = −12→10

### Refinement

**Table:**

Refinement on *F*^2^	Hydrogen site location: mixed
Least-squares matrix: full	H atoms treated by a mixture of independent and constrained refinement
*R*[*F*^2^ > 2σ(*F*^2^)] = 0.029	*w* = 1/[σ^2^(*F*_o_^2^) + (0.0364*P*)^2^ + 0.1566*P*] where *P* = (*F*_o_^2^ + 2*F*_c_^2^)/3
*wR*(*F*^2^) = 0.073	(Δ/σ)_max_ = 0.001
*S* = 1.03	Δρ_max_ = 0.23 e Å^−^^3^
2761 reflections	Δρ_min_ = −0.20 e Å^−^^3^
197 parameters	Absolute structure: Flack x determined using 1097 quotients [(I+)-(I-)]/[(I+)+(I-)] (Parsons *et al.*, 2013).
1 restraint	Absolute structure parameter: −0.4 (4)

### Special details

**Table:**

Geometry. All esds (except the esd in the dihedral angle between two l.s. planes) are estimated using the full covariance matrix. The cell esds are taken into account individually in the estimation of esds in distances, angles and torsion angles; correlations between esds in cell parameters are only used when they are defined by crystal symmetry. An approximate (isotropic) treatment of cell esds is used for estimating esds involving l.s. planes.

### Fractional atomic coordinates and isotropic or equivalent isotropic displacement parameters (Å^2^)

**Table:**

	*x*	*y*	*z*	*U*_iso_*/*U*_eq_	
O1	0.7009 (3)	0.99423 (13)	0.44759 (17)	0.0210 (3)	
O2	0.6482 (3)	1.23008 (14)	0.42123 (17)	0.0236 (4)	
O3	0.9451 (3)	1.26855 (13)	0.68102 (17)	0.0223 (3)	
O4	1.3447 (3)	1.16847 (15)	0.61241 (19)	0.0271 (4)	
O5	1.0920 (3)	1.01394 (12)	0.41337 (15)	0.0200 (3)	
O7	0.4680 (3)	0.86115 (14)	0.24446 (16)	0.0215 (3)	
O8	0.2727 (3)	0.93833 (15)	0.05537 (16)	0.0283 (4)	
N1	0.8644 (3)	0.95057 (15)	0.16816 (17)	0.0160 (3)	
H1A	0.872660	0.899266	0.238389	0.019*	
H1B	1.003891	0.951327	0.132317	0.019*	
C1	0.8227 (4)	1.06402 (17)	0.2287 (2)	0.0189 (4)	
H1C	0.661121	1.087516	0.201456	0.023*	
H1D	0.927252	1.119552	0.191299	0.023*	
C2	0.8665 (4)	1.05974 (17)	0.3875 (2)	0.0182 (4)	
C3	0.8630 (4)	1.17629 (17)	0.4547 (2)	0.0177 (4)	
H3	0.986207	1.222671	0.417043	0.021*	
C4	0.9241 (4)	1.16278 (17)	0.6125 (2)	0.0178 (4)	
H4	0.799149	1.118808	0.652062	0.021*	
C5	1.1540 (4)	1.10136 (18)	0.6441 (2)	0.0205 (4)	
H5	1.173771	1.081867	0.745658	0.025*	
C6	1.1571 (4)	0.99419 (18)	0.5592 (2)	0.0215 (5)	
H6A	1.314355	0.961418	0.570981	0.026*	
H6B	1.048878	0.939529	0.594849	0.026*	
C7	0.6822 (3)	0.91363 (17)	0.0555 (2)	0.0164 (4)	
C8	0.6746 (4)	0.99140 (19)	−0.0720 (2)	0.0202 (4)	
H8A	0.549752	1.047572	−0.069571	0.024*	
H8B	0.824036	1.030727	−0.075537	0.024*	
C9	0.6270 (4)	0.9129 (2)	−0.1969 (2)	0.0270 (5)	
H9A	0.461610	0.891584	−0.210590	0.032*	
H9B	0.672248	0.947623	−0.283720	0.032*	
C10	0.7793 (5)	0.8121 (2)	−0.1546 (2)	0.0295 (5)	
H10A	0.943566	0.827661	−0.167132	0.035*	
H10B	0.728704	0.745231	−0.210171	0.035*	
C11	0.7450 (4)	0.79532 (18)	0.0003 (2)	0.0220 (4)	
H11A	0.888615	0.766805	0.052214	0.026*	
H11B	0.618351	0.741477	0.011014	0.026*	
C12	0.4522 (4)	0.90500 (18)	0.1238 (2)	0.0188 (4)	
H4O	1.347 (5)	1.227 (3)	0.662 (3)	0.032 (8)*	
H1O	0.631 (6)	0.955 (3)	0.390 (4)	0.042 (9)*	
H3O	0.814 (6)	1.296 (3)	0.684 (3)	0.037 (8)*	
H2O	0.553 (6)	1.199 (3)	0.463 (3)	0.037 (9)*	

### Atomic displacement parameters (Å^2^)

**Table:**

	*U*^11^	*U*^22^	*U*^33^	*U*^12^	*U*^13^	*U*^23^
O1	0.0221 (8)	0.0233 (8)	0.0179 (7)	−0.0065 (6)	0.0033 (6)	−0.0029 (6)
O2	0.0212 (9)	0.0230 (8)	0.0263 (9)	0.0047 (7)	0.0009 (7)	0.0015 (7)
O3	0.0165 (8)	0.0229 (7)	0.0272 (9)	0.0015 (6)	0.0009 (6)	−0.0097 (6)
O4	0.0173 (8)	0.0290 (9)	0.0359 (9)	−0.0021 (7)	0.0066 (7)	−0.0129 (8)
O5	0.0191 (8)	0.0227 (8)	0.0180 (7)	0.0022 (6)	0.0014 (6)	−0.0033 (6)
O7	0.0184 (7)	0.0269 (8)	0.0195 (8)	−0.0031 (6)	0.0037 (6)	0.0034 (6)
O8	0.0144 (7)	0.0461 (11)	0.0244 (8)	0.0031 (7)	0.0019 (6)	0.0037 (8)
N1	0.0130 (8)	0.0193 (8)	0.0159 (8)	−0.0007 (7)	0.0021 (6)	0.0008 (7)
C1	0.0215 (10)	0.0173 (10)	0.0180 (10)	−0.0001 (8)	0.0023 (8)	−0.0006 (8)
C2	0.0182 (10)	0.0194 (10)	0.0168 (10)	−0.0008 (8)	0.0012 (8)	−0.0004 (8)
C3	0.0164 (10)	0.0177 (10)	0.0193 (10)	0.0006 (8)	0.0026 (8)	−0.0014 (8)
C4	0.0172 (10)	0.0176 (9)	0.0187 (10)	−0.0011 (8)	0.0020 (8)	−0.0033 (8)
C5	0.0182 (10)	0.0244 (11)	0.0188 (10)	0.0011 (9)	0.0017 (8)	−0.0032 (9)
C6	0.0219 (11)	0.0214 (10)	0.0204 (11)	0.0048 (9)	−0.0018 (8)	−0.0010 (9)
C7	0.0144 (9)	0.0193 (9)	0.0155 (9)	−0.0002 (8)	0.0013 (7)	−0.0009 (8)
C8	0.0202 (11)	0.0227 (10)	0.0177 (10)	−0.0020 (8)	0.0017 (8)	0.0029 (8)
C9	0.0289 (12)	0.0334 (12)	0.0183 (10)	−0.0022 (10)	0.0005 (9)	0.0013 (9)
C10	0.0311 (13)	0.0353 (13)	0.0226 (12)	0.0036 (11)	0.0049 (9)	−0.0055 (10)
C11	0.0239 (11)	0.0202 (10)	0.0216 (10)	0.0019 (8)	0.0009 (8)	−0.0029 (8)
C12	0.0162 (10)	0.0205 (9)	0.0200 (10)	−0.0024 (8)	0.0038 (8)	−0.0028 (8)

### Geometric parameters (Å, º)

**Table:**

O1—C2	1.406 (3)	C3—H3	1.0000
O1—H1O	0.80 (4)	C4—C5	1.528 (3)
O2—C3	1.412 (3)	C4—H4	1.0000
O2—H2O	0.81 (3)	C5—C6	1.520 (3)
O3—C4	1.425 (2)	C5—H5	1.0000
O3—H3O	0.84 (4)	C6—H6A	0.9900
O4—C5	1.423 (3)	C6—H6B	0.9900
O4—H4O	0.85 (3)	C7—C8	1.535 (3)
O5—C2	1.419 (3)	C7—C12	1.547 (3)
O5—C6	1.436 (3)	C7—C11	1.566 (3)
O7—C12	1.269 (3)	C8—C9	1.529 (3)
O8—C12	1.243 (3)	C8—H8A	0.9900
N1—C1	1.504 (3)	C8—H8B	0.9900
N1—C7	1.506 (3)	C9—C10	1.526 (3)
N1—H1A	0.9100	C9—H9A	0.9900
N1—H1B	0.9100	C9—H9B	0.9900
C1—C2	1.524 (3)	C10—C11	1.535 (3)
C1—H1C	0.9900	C10—H10A	0.9900
C1—H1D	0.9900	C10—H10B	0.9900
C2—C3	1.537 (3)	C11—H11A	0.9900
C3—C4	1.533 (3)	C11—H11B	0.9900
			
C2—O1—H1O	111 (2)	C4—C5—H5	108.8
C3—O2—H2O	108 (2)	O5—C6—C5	111.70 (17)
C4—O3—H3O	109 (2)	O5—C6—H6A	109.3
C5—O4—H4O	108 (2)	C5—C6—H6A	109.3
C2—O5—C6	112.75 (15)	O5—C6—H6B	109.3
C1—N1—C7	114.50 (16)	C5—C6—H6B	109.3
C1—N1—H1A	108.6	H6A—C6—H6B	107.9
C7—N1—H1A	108.6	N1—C7—C8	111.15 (16)
C1—N1—H1B	108.6	N1—C7—C12	106.82 (16)
C7—N1—H1B	108.6	C8—C7—C12	114.79 (17)
H1A—N1—H1B	107.6	N1—C7—C11	109.76 (17)
N1—C1—C2	109.85 (16)	C8—C7—C11	105.47 (17)
N1—C1—H1C	109.7	C12—C7—C11	108.79 (17)
C2—C1—H1C	109.7	C9—C8—C7	104.14 (18)
N1—C1—H1D	109.7	C9—C8—H8A	110.9
C2—C1—H1D	109.7	C7—C8—H8A	110.9
H1C—C1—H1D	108.2	C9—C8—H8B	110.9
O1—C2—O5	111.62 (17)	C7—C8—H8B	110.9
O1—C2—C1	112.06 (17)	H8A—C8—H8B	108.9
O5—C2—C1	104.54 (16)	C10—C9—C8	102.65 (19)
O1—C2—C3	107.14 (17)	C10—C9—H9A	111.2
O5—C2—C3	108.97 (17)	C8—C9—H9A	111.2
C1—C2—C3	112.54 (17)	C10—C9—H9B	111.2
O2—C3—C4	112.90 (17)	C8—C9—H9B	111.2
O2—C3—C2	111.32 (18)	H9A—C9—H9B	109.1
C4—C3—C2	108.05 (16)	C9—C10—C11	103.64 (19)
O2—C3—H3	108.1	C9—C10—H10A	111.0
C4—C3—H3	108.1	C11—C10—H10A	111.0
C2—C3—H3	108.1	C9—C10—H10B	111.0
O3—C4—C5	107.48 (17)	C11—C10—H10B	111.0
O3—C4—C3	111.41 (17)	H10A—C10—H10B	109.0
C5—C4—C3	111.20 (17)	C10—C11—C7	105.47 (18)
O3—C4—H4	108.9	C10—C11—H11A	110.6
C5—C4—H4	108.9	C7—C11—H11A	110.6
C3—C4—H4	108.9	C10—C11—H11B	110.6
O4—C5—C6	108.13 (17)	C7—C11—H11B	110.6
O4—C5—C4	111.66 (18)	H11A—C11—H11B	108.8
C6—C5—C4	110.70 (17)	O8—C12—O7	126.9 (2)
O4—C5—H5	108.8	O8—C12—C7	117.89 (19)
C6—C5—H5	108.8	O7—C12—C7	115.22 (18)
			
C7—N1—C1—C2	135.27 (18)	C2—O5—C6—C5	−59.7 (2)
C6—O5—C2—O1	−53.6 (2)	O4—C5—C6—O5	−71.6 (2)
C6—O5—C2—C1	−174.91 (17)	C4—C5—C6—O5	51.0 (2)
C6—O5—C2—C3	64.6 (2)	C1—N1—C7—C8	63.4 (2)
N1—C1—C2—O1	−67.8 (2)	C1—N1—C7—C12	−62.6 (2)
N1—C1—C2—O5	53.2 (2)	C1—N1—C7—C11	179.67 (16)
N1—C1—C2—C3	171.33 (17)	N1—C7—C8—C9	142.64 (17)
O1—C2—C3—O2	−64.5 (2)	C12—C7—C8—C9	−96.0 (2)
O5—C2—C3—O2	174.57 (17)	C11—C7—C8—C9	23.7 (2)
C1—C2—C3—O2	59.1 (2)	C7—C8—C9—C10	−40.9 (2)
O1—C2—C3—C4	60.0 (2)	C8—C9—C10—C11	42.1 (2)
O5—C2—C3—C4	−60.9 (2)	C9—C10—C11—C7	−27.1 (2)
C1—C2—C3—C4	−176.39 (17)	N1—C7—C11—C10	−117.74 (19)
O2—C3—C4—O3	−61.7 (2)	C8—C7—C11—C10	2.1 (2)
C2—C3—C4—O3	174.70 (18)	C12—C7—C11—C10	125.71 (19)
O2—C3—C4—C5	178.39 (18)	N1—C7—C12—O8	139.8 (2)
C2—C3—C4—C5	54.8 (2)	C8—C7—C12—O8	16.1 (3)
O3—C4—C5—O4	−52.0 (2)	C11—C7—C12—O8	−101.8 (2)
C3—C4—C5—O4	70.2 (2)	N1—C7—C12—O7	−41.9 (2)
O3—C4—C5—C6	−172.52 (17)	C8—C7—C12—O7	−165.61 (19)
C3—C4—C5—C6	−50.3 (2)	C11—C7—C12—O7	76.5 (2)

### Hydrogen-bond geometry (Å, º)

**Table:**

*D*—H···*A*	*D*—H	H···*A*	*D*···*A*	*D*—H···*A*
N1—H1*B*···O8^i^	0.91	1.80	2.704 (2)	174
N1—H1*A*···O3^ii^	0.91	2.00	2.784 (2)	143
N1—H1*A*···O1	0.91	2.59	2.980 (2)	107
O4—H4*O*···O3	0.85 (3)	2.41 (3)	2.746 (2)	105 (2)
O4—H4*O*···O7^iii^	0.85 (3)	2.09 (3)	2.845 (2)	149 (3)
O1—H1*O*···O7	0.80 (4)	1.97 (4)	2.769 (2)	178 (3)
O3—H3*O*···O7^iv^	0.84 (4)	1.99 (4)	2.796 (2)	162 (3)
O2—H2*O*···O4^v^	0.81 (3)	2.00 (3)	2.765 (2)	159 (3)

Symmetry codes: (i) *x*+1, *y*, *z*; (ii) −*x*+2, *y*−1/2, −*z*+1; (iii) −*x*+2, *y*+1/2, −*z*+1; (iv) −*x*+1, *y*+1/2, −*z*+1; (v) *x*−1, *y*, *z*.
